# Microbial Diversity and Community Composition of Duodenum Microbiota of High and Low Egg-Yielding Taihang Chickens Identified Using 16S rRNA Amplicon Sequencing

**DOI:** 10.3390/life12081262

**Published:** 2022-08-18

**Authors:** Haiyin Han, Yingjie Sun, Yekai Fan, Hui Zhang, Junqi Yang, Runqing Chi, Yahui Gao, Jiannan Liu, Kaiyang Li, Wenting Li, Yufang Liu

**Affiliations:** 1 School of Life Sciences and Food Engineering, Hebei University of Engineering, Handan 056021, China; 2 School of Landscape and Ecological Engineering, Hebei University of Engineering, Handan 056021, China; 3 Beijing General Station of Animal Husbandry, Beijing 100107, China; 4 College of Animal Sciences and Technology, Henan Agricultural University, Zhengzhou 450046, China

**Keywords:** Taihang chicken, high-throughput sequencing, bacterial diversity, egg-laying performance, function prediction

## Abstract

The duodenum is an important digestive organ for poultry and houses a variety of microbes that help chickens to enhance nutrient absorption and improve production. To evaluate the characteristic of gut microbiome, duodenum content samples from 42-week-old native Taihang chickens with high (H) and low (L) egg-yielding were collected for 16S rRNA amplicon sequencing analysis. Consequently, 1,361,341 sequences were clustered into 2055 OTUs, with percentages of affiliation of 96.50 and 57.30% at phylum and genus levels. *Firmicutes*, *Proteobacteria*, *Cyanobacteria* and *Bacteroidetes* were the dominant phylum, with a lower ratio of *Firmicutes/Bacteroidetes* in H group than in L group (*p* < 0.05). At genus level, overrepresentation of *Bacteroides*, *Faecalibacterim*, and *Enterococcus* and underrepresentation of *Romboutsia* were found in H group. No significant difference in overall diversity of microbiota was observed between two groups. LEFSe analysis revealed *Enterococcus* was significantly enriched in H group. Importantly, *Enterococcus* and *Lactobacillus* were negatively correlated. Functional prediction analysis showed the proportion of microbiota involved in the metabolism process was the highest and enriched in H group. Differences in microbiota composition between the two groups, which may be related to intestinal function difference, also provide promising biomarkers for improving laying hen production.

## 1. Introduction

The world population is rapidly growing and will reach 9.6 billion in 2050, and this growth continues to necessitate a larger supply of food, especially proteins [1]. As a significant supply of animal protein, worldwide consumption of poultry eggs has increased greatly in the last few years [2]. Correspondingly, chickens, the world’s largest poultry species, are relatively inexpensive to produce and have high feed conversion ratios [3]. The Taihang chicken is a well-known native breed used for both egg and meat production in Hebei province, China. It has outstanding traits like strong disease resistance, high feed tolerance, cold resistance, and other excellent traits. However, poor production performance is the typical characteristic of Taihang chickens, which are commonly free-range in remote mountainous areas of northern China [4]. Therefore, it is urgent to improve the productivity of the Taihang chicken, especially the egg production trait.

The term “microbiota” refers to all microorganisms that can be present in a certain ecosystem, including bacteria, viruses, and funguses [5,6]. The intestinal microbiota and their host have a vital and beneficial relationship. With further understanding of the community structure and functional capacity of the intestinal microbiota, it is possible to identify the connections between microbiota functions and the host’s physiology and metabolism. According to previous research, the bacteria begin to inhibit the gastrointestinal tract of chickens on the day after they hatch [7]. The gut microbes play vital role in improving chicken nutrient availability, absorptive capacity, and thus the productivity [8]. Numerous investigations have confirmed the pronounce influence of the gut microbes on the productive performance of chickens [9,10]. For example, egg production could be increased by giving laying hens a 0.6% *Lactobacillus* supplement in their diet [9]. The duodenum is an important part of the small intestine and is a vital digestive organ in poultry. The main function of the duodenum is to be the region where most of the glucose and other nutrients are absorbed [11,12,13]. Different areas of the intestine harbor different microflora. The intestinal microbes of chickens are still being studied, and the *Firmicutes* (30–50%) and *Bacteroidetes* (10–50%) phyla comprise the majority of the duodenal microorganisms [14,15]. Moreover, chicken duodenum is dominated by *Lactobacillus* (account for nearly 99% among some chicken breeds), *Streptococcus* and coliforms at the genus level [16]. Amplicon sequencing, which targets the microbe 16S rRNA gene, has been by far the most widely adopted sequencing technique in microbiome research. Reports have been made on the analysis of the composition of the intestinal bacteria of broiler chickens [17], Dagu chickens [18], Naked Neck chickens [19] and egg-laying hens [20] using the 16S rRNA gene amplicon sequencing.

Egg production of Taihang chickens varies considerably under the same genetic background, feed and management conditions. The link between the intestinal microbial communities and egg production capacity in hens is still unclear. Hence, the purpose of the present work was to assess the association between egg production and intestinal microbiota in selected 42-week-old high (H) and low (L) egg-laying Taihang chickens using 16S rRNA gene amplicon sequencing of their duodenal microbiota. Differences in bacteria communities between groups of chickens with different productivity were analyzed. This study provides a reference for further research on Taihang chickens and may improve chicken husbandry practices to improve egg production.

## 2. Materials and Methods

### 2.1. Sample Collection

In this study, 800 female Taihang chickens were individually housed with free access to feed and water under the same feed nutrition, management methods, and environmental conditions. All chickens were fed the basic diet with 11.02 MJ/kg of metabolizable energy (ME); 15.76% of crude protein, 3.32% of Calcium; 0.57% of total phosphorus. The composition, nutritional level, and the precise mineral-vitamin concentrations of the basic diet were shown in Table 1. The number of eggs laid were recorded from age at first lay. After recording egg laying for 30 consecutive days, the egg production of chickens with the healthy appearance was continuously recorded. The chickens with health, stable laying, well colored and similar body weight were reserved at 42 weeks of age at the peak laying period. Then, based on the 42 weeks of age egg-laying number, twenty Taihang chickens were randomly selected and divided into high (H, *n* = 10, average egg-laying number was 68.33 ± 0.40) and low (L, *n* = 10, average egg-laying number was 48.12 ± 0.40) groups (*p* < 0.05). The chickens were euthanized, the duodenums were dissected, and the luminal contents were sampled as soon as possible. To ascertain the uniformity of samples between chickens, a 5 cm length of the duodenal fixed portion was selected from each individual for sampling. The two ends of the selected duodenal portion were held and fixed with forceps. The selected intestinal segment was cut off, the forceps was removed at the end near the cloacal, and squeezed appropriately to allow the contents to flow into the storage tube. The duodenum content samples were transferred directly to liquid nitrogen and then placed in a −80 °C refrigerator.

### 2.2. Microbiota DNA Isolation and Amplicon Generation

Total DNA was isolated from the duodenal content samples with the CTAB/SDS method and preserved at −20 °C before subsequent studies. DNA concentration and purity were monitored by the NanoDrop 2000 spectrophotometer (Thermo Scientific, Wilmington, DE, USA) and 1% agarose gel electrophoresis, respectively. DNA samples were diluted to 1 ng/μL with sterile water based on its concentration.

PCR was used to amplify the 16S rRNA gene V4 fragment with the barcode primers (515F-806R). Each PCR reaction was performed in a 30 μL reaction, which included 15 μL Phusion^®^ High-Fidelity PCR Master Mix (New England Biolabs, Ipswich, MA, USA); 0.2 μM forward and reverse primers, and approximately 10 ng template DNA. Thermal cycling comprises a preliminary denaturation at 98 °C for 1 min, accompanied by 30 cycles of denaturation at 98 °C for 10 s, annealing at 50 °C for 30 s, and extension at 72 °C for 30 s. Finally, 72 °C for 5 min. PCR efficiency was detected on 2% agarose gel through visual inspection. The products of PCR were blended in equal density ratios and subjected to further extraction using the GeneJET^TM^ Gel Extraction Kit (Thermo Scientific, Waltham, NY, USA).

### 2.3. Libraries Construction and Raw Data Analysis

Sequencing libraries were constructed with Ion Plus Fragment Library Kit 48 rxns (Thermo Scientific, DE, USA) according to the producer’s instructions. The quality of the sequencing libraries was evaluated using Qubit@ 2.0 Fluorometer (Thermo Scientific, Waltham, DE, USA). Finally, the Ion S5^TM^ XL platform was used for sequencing the library and 400 bp/600 bp single-end reads were obtained.

The generated short reads were assigned to samples according to their distinctive barcode and truncated via cut-off barcodes and primer sequences. Based on Cutadapt [21] (V1.9.1, http://cutadapt.readthedocs.io/en/stable, accessed on 25 April 2022) quality controlled process, the raw tags were quality filtered with certain filtering conditions to gain high quality clean reads. Then, comparing the obtained reads with the reference database (Silva database, http://www.arb-silva.de/, accessed on 26 April 2022) [22] using UCHIME method (UCHIME Algorithm, http://www.drive5.com/usearch/manual/uchime_algo.html, accessed on 26 April 2022) [23] to detect and remove the chimera sequences [24]. After that, clean reads were eventually acquired.

### 2.4. Operational Taxonomic Unit (OTU) Clusters and Species Annotation

Sequences analysis was carried out using Uparse software (Uparse v7.0.1001, http://drive5.com/uparse/, accessed on 28 April 2022) [25]. Subsequently, sequences with a similarity score more than 97% were classified into the same OTUs. Representative sequences for all OTUs were filtered for further annotation. Finally, the Silva Database (https://www.arb-silva.de/, accessed on 28 April 2022) [22] was applied depended on RDP classifier (Version 2.2, http://sourceforge.net/projects/rdp-classifier/ accessed on 28 April 2022) [26] method to label taxonomic data for all representative sequences. 

According to the MUSCLE software (Version 3.8.31, http://www.drive5.com/muscle/, accessed on 29 April 2022) [27], multiple sequence alignment were carried out to examine the phylogenetic relationships of various OTUs and differences of the dominant species in various samples (groups). Then, the OTUs abundance data was normalized with a standard of sequence number matching to the sample with the least sequences. Finally, based on the output normalized data, further analyses of alpha and beta diversity were conducted.

### 2.5. Alpha Diversity Analysis

Alpha diversity was analyzed and displayed with QIIME software (Version 1.7.0) and R software (Version 2.15.3), respectively. Sequences with 97% similarity were clustered and Rarefaction curves were generated with QIIME software. In the rarefaction curve analysis, rarefaction to 43,420 sequences was conducted for all samples. The Boxplots were constructed with BoxPlotR software [28]. Metrics of alpha diversity included Observed_species and Shannon’s indices.

### 2.6. Beta Diversity Analysis

The Beta diversity analysis was performed to assess species differences in samples. Beta diversity analysis of weighted and unweighted unifrac distances was evaluated with QIIME (Version 1.7.0). WGCNA package, stat packages and ggplot2 package in R software (Version 2.15.3) were used to generate principal-coordinate analysis (PCoA) plots with unweighted and weighted UniFrac distances. Moreover, Principal component analysis (PCA) was performed using FactoMine R package and ggplot2 package in R software (Version 2.15.3). The following downstream diagrams were produced with the R package. Non-Metric Multidimensional Scaling method (NMDS) was chosen to visualize the dissimilarity matrix between samples which was computed through Bray-Curtis distances. Unweighted Pair-group Method with Arithmetic Means (UPGMA) Clustering, a hierarchical clustering approach for interpreting distance matrix, was carried out through average linkage and was performed with QIIME (Version 1.7.0). ANOSIM analysis on Bray-Curtis distances was done with R vegan anosim. A percentage similarity analysis (Simper) was used to estimate the dissimilarity between samples. SIMPER was performed with the R vegan simper, a data pretreatment by the Bray-Curtis similarity coefficient.

### 2.7. Specific Biomarkers 

To identify chicken egg-laying performance specific biomarker at multiple taxonomical levels, the bacterial abundance profile was analyzed using linear discriminant analysis (LDA) effect size (LEFSe). The bacterial abundance curves were generated at a classification level from phylum to the species in the present investigation. LDA scores of >4 (on a log_10_ scale) were used as thresholds.

### 2.8. Network Construction 

According to the species abundance, calculate the correlation coefficient values (Spearman’s correlation coefficient SCC) of each genus, get the correlation coefficient matrix, and set the criteria for filtration: (a) set the threshold value (>0.6) to screen out the poorly associated connections; (b) filter out node self-joining; and (c) eliminate connections with node abundance values smaller than 0.005%. According to the relevant value of filtration, taking bacteria as nodes and values as edges, graphviz-2.38.0 was used to draw network diagrams.

### 2.9. Function Prediction Analysis

16S rRNA gene sequence of the prokaryotic whole genome was first obtained from the KEGG database. Second, a correlation matrix was created by matching the 16S rRNA gene sequence with the SILVA SSU Ref NR database via the BLASTN method (BLAST Bit score > 1500). Thirdly, the SILVA database function annotation was achieved by mapping the prokaryotic whole genome functional information of the KEGG database annotated by UProC and PAUDA to the SILVA database. Based on the minimum 16S rRNA sequence similarity, Tax4Fun functional prediction was done through the nearest neighbor approach. Finally, in order to gain the functional annotation information, the sequenced reads were clustered from the OTUs using the SILVA database sequence as reference sequence.

### 2.10. Statistical Analyses

The student’s *t*-test was used to determine the significance of differences in egg production levels between H and L groups, and the *t*-test and Wilcoxon sum-rank test were used to determine alpha diversity and beta diversity of the microbe. The detailed analysis was carried out using SPSS 22.0 (Chicago, IL, USA). In the LEfSe analysis, the Wilcoxon sum-rank test was conducted to confirm biological differences between different groups while the non-parametric factorial Kruskal–Wallis sum-rank test was employed to find species with notable differential abundances. Then, LDA score histograms were used to calculate the effect size for identifying the particular taxa. Statistical significance was defined as *p* value < 0.05 and *p* < 0.01.

## 3. Results

### 3.1. 16S rRNA Sequencing Date

**Figure 1:**
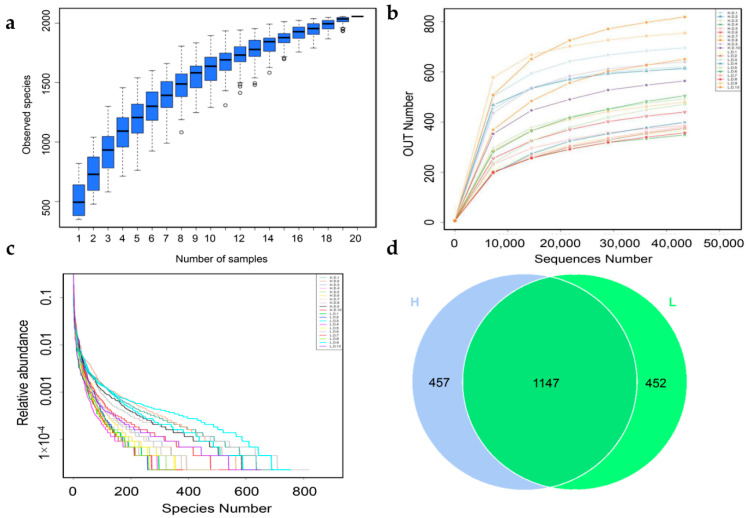
Sample abundance analysis. The species accumulation boxplot (**a**); rarefaction curves (**b**); and Rank abundance curve (**c**) were based on the OTU number; (**d**) a Venn diagram of the OTUs for H and L group.

To characterize the intestinal microbiota composition of Taihang chickens with high (H) and low (L) egg production capacity, 16S ribosomal RNA (rRNA) sequence was performed on all twenty samples. The flat trends span and smoothness of the species accumulation boxplot confirmed the sample size was sufficient (Figure 1a). Similarly, the rarefaction curve indicated that there were adequate reads to cover the whole microbial community (Figure 1b). Moreover, the rank abundance curve reflected the diversity and uniformity of microbiota among the samples (Figure 1c). 

As a result, a total of 1,361,341 clean sequences were obtained (Table S1). With 97% sequence similarity, 2056 OTUs were obtained. After leveling, 2055 OTUs were annotated to the SILVA 132 database, with percentages of affiliation of 96.50 and 57.30% at the phylum and genus levels, respectively (Table 2). More detail information about OTUs among samples was shown in Figure S1. As shown in Figure 1d, 457 and 452 OTUs were observed in H and L groups respectively, and 1147 common OTUs shared across the two groups (Figure 1d). 

### 3.2. Microbiome Taxonomic Profiles

**Figure 2:**
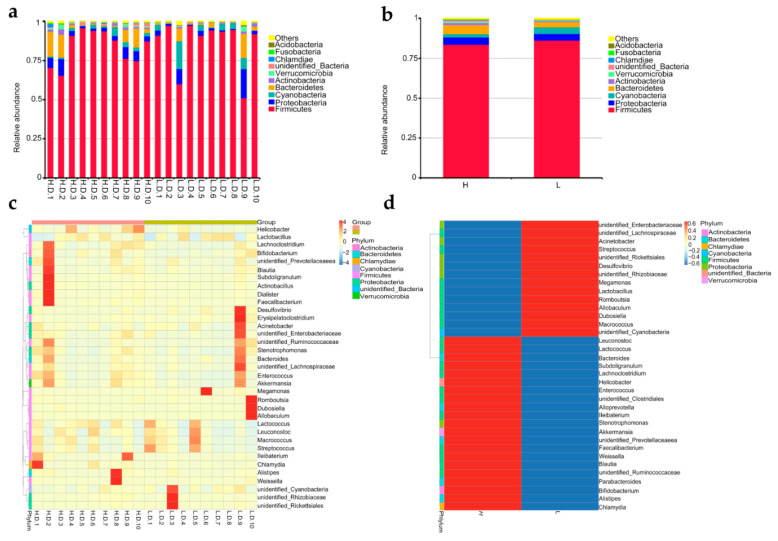
Comparative of duodenum microbiome abundance at phyla and genus level. Differences in the relative abundance of top 10 microbial phyla among samples (intragroup) (**a**); and between H and L groups (**b**); heatmap hierarchical cluster analysis based on the 35 most abundant genera among samples (intragroup) (**c**); and between H and L groups (**d**). The date represents the average percentage of all sequences that have been detected, and each bar represents the average of a sample or a group. The relative abundance was drawn intuitively from red to blue; red represented the highest abundant (max = 4), while blue (min = −4) represented the lowest abundant.

The duodenal microbiome abundance was assessed to gain the differences in microbial composition. The results indicated that *Firmicutes*, *Proteobacteria*, *Cyanobacteria*, and *Bacteroidetes* were the main phyla of microbes, of which *Firmicutes* were the most abundant phylum (Figure 2a). The abundance of the other microbial phyla was noticeably lower than that of the major phyla. Although there was no taxonomic variation among all samples, a comparison of the disparities in the abundance of bacteria taxa between H and L groups was shown in Figure 2b. For instance, there was a lower relative abundance of *Firmicutes* and *Cyanobacteria* in H group than that in L group, while that of *Bacteroidetes* was higher in H group than that in L group. As a result, the *Firmicutes/Bacteroidetes* ratio was obviously lower in H group compared to L group (*p* < 0.05).

To further investigate the relative abundance of the predominant bacteria, heatmaps were drawn for 35 genera with the highest abundant (Figure 2c,d). The stable genera among all samples changed to varying degrees. The populations of a total of 21 genera were apparently higher in H group, including *Enterococcus*, *Weissella*, *Lactococcus*, *Faecalibacterium*, *Akkemansia*, *Bacteroides* and others. However, higher abundances of other 14 genera including *Lactobacillus*, *Romboutsia* and *Streptococcus* were observed in L group.

**Figure 3:**
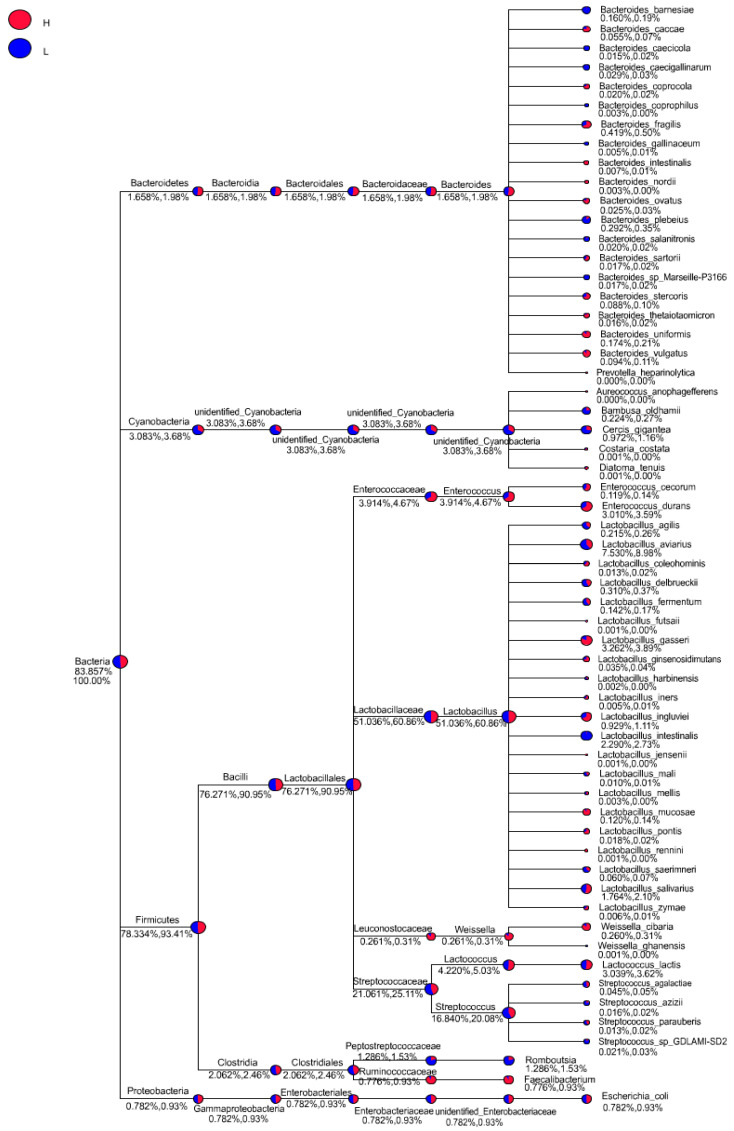
In-depth comparative analysis of genus with phylogenetic tree. Each circle on the phylogenetic tree node was scaled logarithmically to indicate the relative abundance of each genera with a pie chart distinguishing between H and L groups.

Meanwhile, genus-level phylogenetic comparison tree suggested in-depth variations in microbiota community (Figure 3). A percentage increase in several genera, including *Enterococcus*, *Weissella*, *Lactococcus* and *Faecalibacterium*, in H group was noted. However, a percentage increase in other several genera, including *Romboutsia* and *Streptococcus*, in L group was also noted. Moreover, a noticeable increase in class *Clostridia* belong to phyla *Firmicutes* was found attributable to genera *Romboutsia*.

**Figure 4:**
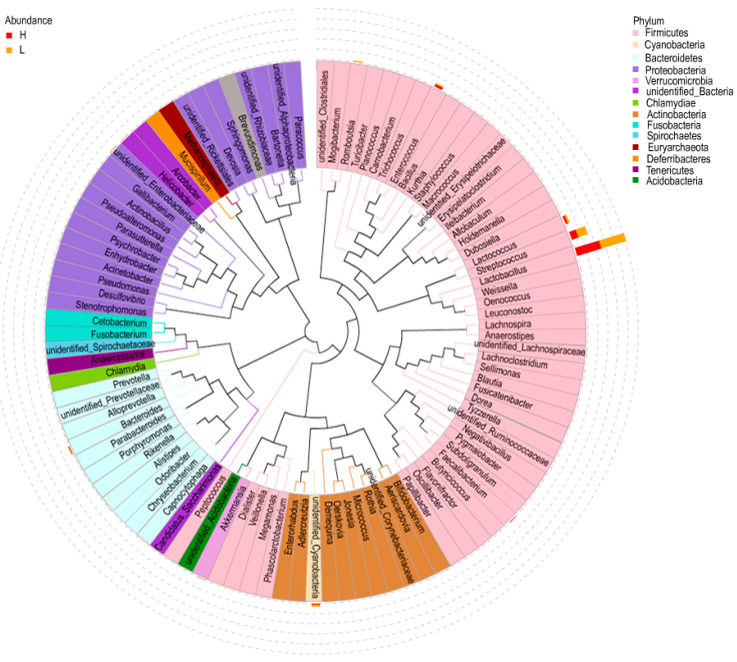
The distribution and representation of the top 100 genera. Bar charts indicated the relative abundance of the genera. The innermost clades and labels were colored by phylum.

Furthermore, the distribution and representation of the top 100 genera were visualized in Figure 4. A total of 6 genera were observed in relatively high abundance in both H and L group, including *Lactobacillus* and *Streptococcus*, *Lactococcus*, *Enterococcus*, *Unidentified-Cyanobacteria*, *Bacteroides*. Noteworthy, the *Enterococcus* and *Bacteroides* were in higher abundance in H group compared to that in L group. Differences in the relative abundance of other genera were evident, although they were at relatively low levels. For example, *Faecalibacterium* was highly abundant in H group, while *Romboutsia* was highly abundant in the L group. The results presented above indicated that the abundances of genera *Bacteroides*, *Enterococcus*, and *Facecalibacterium* were apparently higher in H group, while *Romboutsia* was more abundant in L group (*p* < 0.05).

### 3.3. Bacterial Diversity Analysis

**Figure 5:**
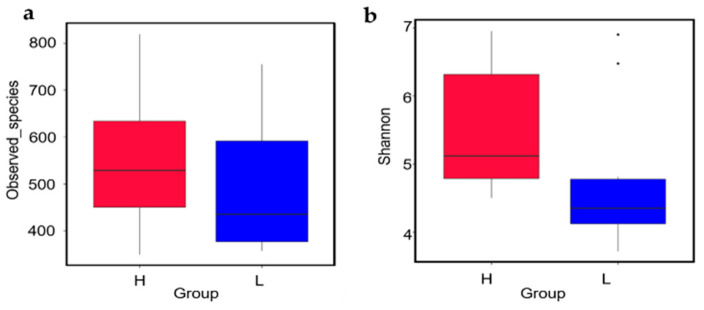
The evaluation of microbial alpha diversity. Alpha diversity in H and L groups (*n* = 10 per group) was estimated using Observed species richness indices (**a**); and Shannon diversity indices (**b**); the median, quartiles, extreme values of the data were displayed on Box plots. Differences in alpha diversity were estimated with the Wilcox test, *p* > 0.05.

To evaluate the structure variation of microbiome between different groups, two alpha diversity indexes, which included Observed species and Shannon, were analyzed. By Wilcoxon test, both Observed species (*p* = 0.3930) and Shannon (*p* = 0.0630) in H group were higher compared to that in L group, but there was no statistical difference observed between the two groups (Figure 5). 

**Figure 6:**
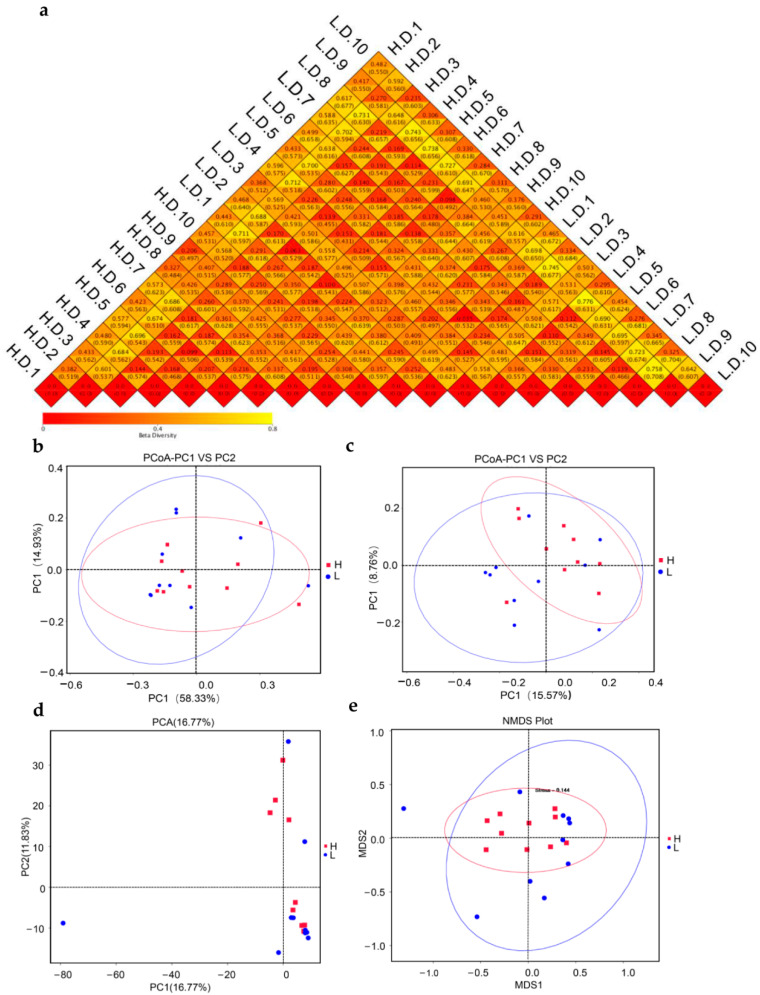
Comparative analysis of the beta diversity: (**a**) heatmap of Beta diversity indices. The difference coefficient between H and L groups was indicated by the number in each square. The disparity in species diversity decreased with decreasing difference coefficient. The upper and lower numbers in the same square stand for the weighted and Unweighted UniFrac distances (mean ± SEM), respectively. Principal coordinate analysis (PCoA) figure based on the weighted UniFrac distance (**b**); and unweighted UniFrac distance (**c**) was drawn; (**d**) principal Coordinate Analysis (PCA) showed the similarities between the two groups; and (**e**) the nonmetric multidimensional scaling (NMDS) analysis revealed differences in microbiome communities based on the Bray-Curtis distance. Red symbols stood for biological replicates within H group, and blue symbols within L group.

To assess the dissimilarity of microbiome structure diversities (beta-diversity) between H and L groups, the weighted UniFrac and unweight UniFrac were calculated (Figure 6a). There was no statistical difference between the two groups based on the PCoA analysis (Figure 6b,c). Similarly, the PCA analysis showed no difference in clustering in H and L groups. Likewise, the NMDS representation did not indicated the microbiota composition clustered by group (Figure 6e). However, the dispersion of samples in L group indicated that the intestinal microbiota differed significantly in this group (Figure 6c–e). 

### 3.4. Bacterial Cluster Analysis

**Figure 7:**
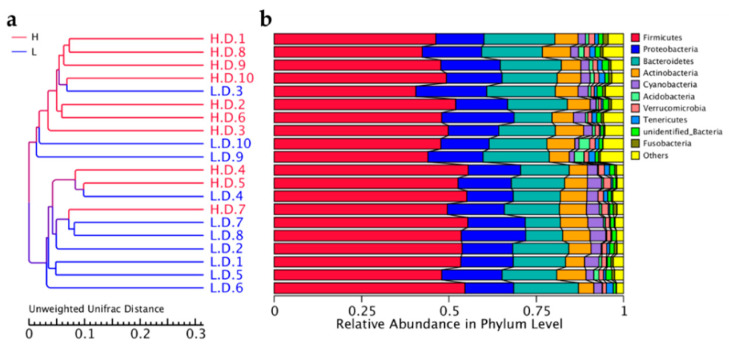
Hierarchical clustering analysis. Dendrogram of Unweighted UniFrac UPGMA cluster analysis (**a**) and the relative abundances of duodenal microbial phyla for all samples (**b**).

The similarity of duodenal microbial community of H and L groups was evaluated using UPGMA hierarchical cluster analysis. The results were presented in terms of the relative abundance of duodenal microbial phyla. It demonstrated that the 10 samples in H or L group clustered well (Figure 7a). Additionally, the clustering results showed phylum *Firmicutes*, *Proteobacteria*, *Cyanobacteria* and *Bacteroidetes* dominated among samples, with *Firmicutes* were the most abundant phylum (Figure 7b).

**Figure 8:**
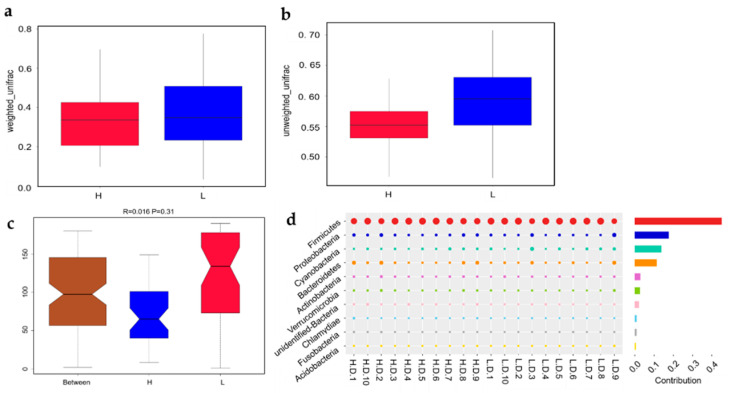
Statistical analysis of bacterial composition differences between groups. Beta diversity indexes of weighted (**a**) and unweighted (**b**) unifrac distance indicated the variance in the duodenal microbiota. The quartiles, median, and extreme values were displayed on the box plots. (**c**) Anosim analysis of Bray–Curtis distance (R = 0.016, *p* = 0.31) showed the difference in the microbiota communities between H and L groups. (**d**) Simper analysis of Bray–Curtis distance verified ten phylum with highest contributions to bacterial dissimilarity and dominance between H and L groups.

According to the beta diversity indices of (un)weighted UniFrac method, there was no significant variation in species composition between H and L groups (Figure 8a,b). However, the ANOSIM statistical analysis (R = 0.016, *p* = 0.31) indicated the variation between groups were larger than that within group (Figure 8c). Although the *p*-value was less distinct, the R-value were greater than zero, indicating potential difference between H and L groups. So, the Simper analysis was employed to further assess the variation in bacterial community composition between H and L groups. The results indicated that 4 phyla, including *Firmicutes*, *Proteobacteria*, *Cyanobacteria* and *Bacteroidetes*, contributed mostly to bacterial dissimilarity and dominated among samples (Figure 8d). The Simper analysis was in agreement with the UPGMA and microbiota composition analysis in this investigation. The analysis above suggested that the microbial composition of H group was same as that of L group.

### 3.5. Linear Discriminate Analysis (LDA) Effect Size (LEfSe) Analysis

**Figure 9:**
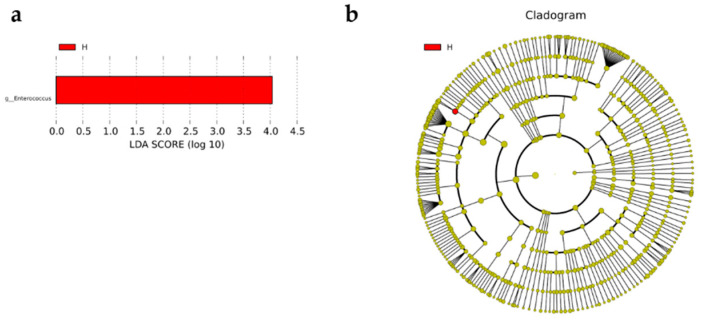
Different microbial composition and development of biomarkers. (**a**) Linear discriminant analysis (LDA) effect size (LEfSe), with a LDA threshold value ≥ 4.0, was used to evaluate the interaction of particular microbiota taxa with H and L group. (**b**) Cladogram showed differently abundant taxa of the duodenal microbiota between H and L groups with the respective cladograms from phylum to species level abundance. Red denotes taxa abundant in H group, and green denotes taxa abundant in L group.

The LEfSe analysis was carried out to identify differential enrichment of microbiota features between H and L groups. By setting LDA score greater than 4.0, genera *Enterococcus* was significantly enriched in H group. However, no bacteria were found to be enriched in L group (Figure 9).

### 3.6. Network Analysis

**Figure 10:**
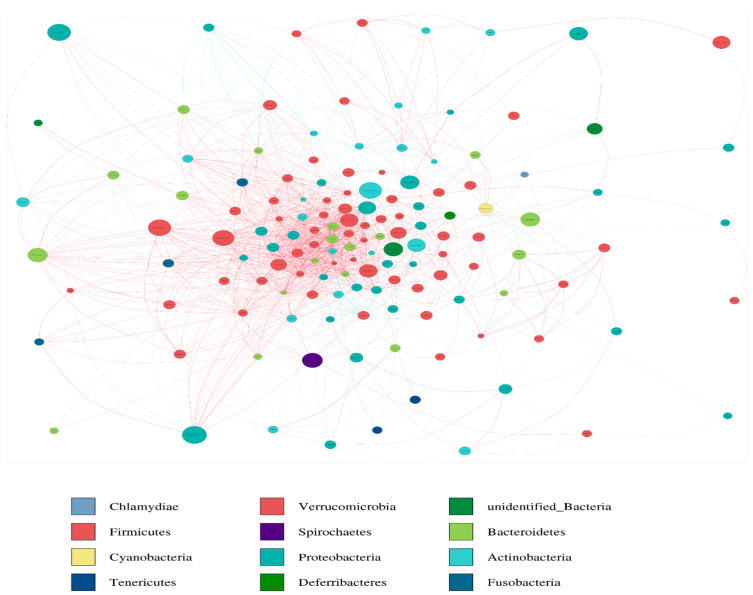
The genus-level microbiome Spearman’s correlation network. The red line denotes substantial positive relation (*p* < 0.05) whereas the blue line reflects notable negative relation (*p* < 0.05).

Correlation among the duodenal microbiota were calculated with Spearman’s correlations. The top 100 abundant taxa among all samples were selected at the genus level. Most genera found in the duodenum were positively related, and a minority were negatively related (Figure 10). The occurrence of genera *Enterococcus* exhibited a negative correlation with *Lactobacillus*. Surprising, *Lactobacillus* exhibited a negative relation with majority of the genera.

### 3.7. Predict Functions of the Microbial Community

**Figure 11:**
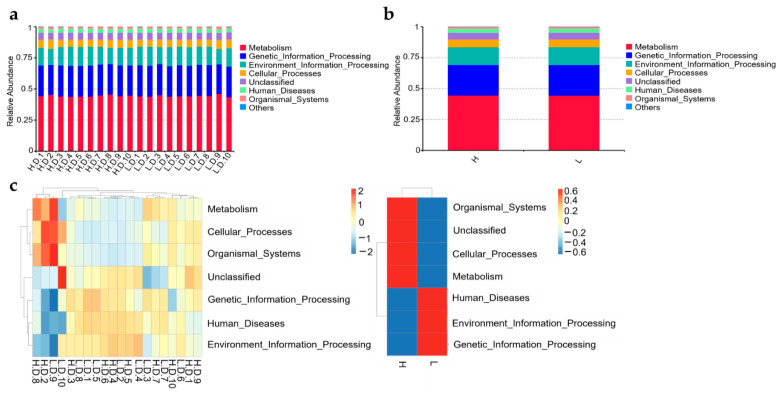
Functional prediction analysis. The relative abundance of Tax4Fun Functional taxa was analyzed among samples (intragroup) (**a**); and between H and L groups (**b**); (**c**) Tax4Fun function annotation clustering heat map. In the microbiota metabolism pathway, red denotes higher enrichment while blue denotes lower enrichment.

The function of the duodenal microbiome was predicted using Tax4Fun analysis. Then, the Kyoto Encyclopaedia of Genes and Genomes (KEGG) level 1 classification system was used to divide the predicted metabolic pathways into six functional groups. The results showed that metabolism, genetic information processing, and environmental information processing were the most abundant functional pathways. There was no significant discrimination in the relative enrichment of different metabolic pathways (Figure 11a,b). 

Tax4Fun function annotation clustering heat map used the top 35 functional categories and showed the cluster at level 1 of functional differences (Figure 11c). In H group, 3 pathways were significantly increased and showed the following functional information: Metabolism, Cellular processes, and Organismal systems. In L group, there was a significant increase of three pathways with functional information on Human disease, environmental information processing and Genetic information processing (Figure 11c). Altogether, the result suggested that there were differences in the metabolic functions of duodenum microbiota of H and L group chickens and therefore deserves further investigation.

## 4. Discussion

Diverse microbiota from the digesta tract have an important impact on the function of host [29]. With the advance and quick progress in metagenome technology, the gut microbiota composition has been studied in several species [30,31], including chicken [32]. Potential associations between the gut bacteria and egg-laying capacity have been demonstrated in laying chicken [9]. In this study, the duodenal bacteria communities were compared between different egg-yielding Taihang chickens via 16S rRNA gene amplicon sequencing. Here, we present a comparative gut microbiota compositions, diversity, and functions analysis of the chickens with different performance. The comparison of microbiota profiles between different performance chickens can offer useful recommendations on the link between microbiome composition and egg production.

Food is mostly digested, absorbed, and fermented in the small intestine, which is made up of the duodenum, jejunum, and ileum. Numerous internal microbial found in the duodenal may have an impact on the health and performance of chickens [33]. Consequently, we analyzed the relationship between duodenal microbial profile and egg production of Taihang chicken. The gut microbiota community is an ecosystem, which made up of many different types of microbes. The interplay of microbe can have a significant impact on environment adaption of host [34]. In our investigation, microbes were groups into operational taxonomic units (OTUs), which were generated by randomly grouping all sequencing reads with an identity of less than 97%. The current study revealed more specific OTUs in H group compared to that in L group, indicating that the H group chickens possess higher diversity of duodenal microbiota.

The investigation of the intestinal microbial composition revealed that these phylum have a distinct dominant pattern. That is, the chicken duodenum was dominated by *Firmicutes*, *Proteobacteria*, *Cyanobacteria* and *Bacteroidetes*, with *Firmicutes* being the most prevalent phyla. Similar findings were discovered in earlier reports [17,35,36]. Noteworthy, the proportions of the microbiota taxa differed between the H and L groups. This study revealed that the proportion of *Bacteroidetes* was relatively higher in the H group, while *Firmicutes* and *Cyanobacteria* in the L group were markedly higher. Previous studies have suggested that *Firmicutes* are associated with energy absorption from nutrition [37]. The phylum *Bacteroidetes* are primarily experts at dissolving proteins and carbohydrates into more basic molecules [38,39], and higher body weight was linked to a reduced abundance of *Bacteroidetes* [40,41]. Moreover, the phyla *Firmicutes* were more prevalent in fat people than in lean people, whereas *Bacteroidetes* were the opposite [41]. Furthermore, it has been demonstrated that the *Firmicutes/Bacteroidetes* ratio plays a crucial role in indicating the status of the intestinal bacteria, and an increase in this ratio has been linked directly to better growth performance [18,42]. Therefore, the lower ratio of *Firmicutes/Bacteroidetes* in the duodenum of Taihang chickens was likely to be related to the lower weight gain, which may result to higher egg production performance. However, the direct effect of the reduced ratio of *Firmicutes/Bacteroidetes* on the body weight needs further validation.

Health status and microbiota alpha diversity were related, and it was found that high alpha diversity was linked to excellent health [43]. In this study, an increasing trend in alpha diversity indices was observed in the H group. According to alpha diversity indices, there was a higher microbial diversity in the high egg-yielding chickens. This outcome was in agreement with the distribution of ordinary and distinctive microbiota OUTs. Moreover, the Shannon indices range was formerly observed to be 4–5 in poultry [44], but 7–9 in rabbits [45], goats [46]) and swine [47]. In this experiment, the Shannon index in the H group was more than 6, which was marginally higher than that in the L group (less than 5), suggesting that the alpha diversity within the duodenal microbiota of H group may affect the egg production. Our data suggested that the egg production of Taihang chickens was related to the variation of gut bacteria composition.

Beta diversity analyses revealed the discrepancies in the microbe profile related to egg production. With the purpose of quantifying the spatial associations of intestinal microbes, uniFrac distances between individuals were determined. Usually, the weighted unifrac distances was determined based on the relative proportion of microbiota to evaluate the community structure. Meanwhile, the unweight unifrac distances was determined based on the presence or absence of microbiota to evaluate the community membership. Hence, the principal coordinates analysis (PCoA) was performed based on weight and unweight uniFrac distances to obtain a general understanding of duodenal microbiota relationships and structure. However, the analyses did not show a difference in microbiota composition across the groups, indicating that the H and L groups shared the similarly microbiota. Similarly, highly similar microbial compositions are indicative of closeness in terms of distance in a PCA diagram. Likewise, statistical analysis using NMDS illustrated the microbial structure did not differ between the H and L groups. Noteworthy, the ANOSIM statistical analysis indicating the potential differences between the two groups. So, according to the Simper analysis, we found 4 phyla, including *Firmicutes*, *Proteobacteria*, *Cyanobacteria* and *Bacteroidetes*, contributed mostly to bacteria dissimilarity and dominance between the H and L groups. The analysis above was agreeable with the results of intestinal microbiota composition.

In the present study, several microbes genera were detected differentially among individuals and between different groups. *Bacteroides* has reportedly been linked to reducing obesity through the production of secondary metabolites [48]. For instance, the proportionate-producing *Bacteroides* species exhibit polysaccharide degradation abilities for nonstarch polysaccharides [49,50]. The Facultative anaerobic *Enterococcus* has been widespread in human and animal digestive tract as well as in nature. Air ingestion, transfer of oxygen from host tissues, and oxygenation through pancreatic and biliary secretions all have an impact on the proportion of *Enterococcus* in anterior digestive tract [51]. It has been demonstrated that *Akkermansia*, a member of phylum *Verrucomicrobia*, was helpful for enhancing obesity and glucose tolerance [52]. *Akkermansia* can be employed a prospective bacteria to enhance chicken performance because it has been considered as a new functional microbe with probiotic qualities. Moreover, short-chain fatty acids-producing microbe *Faecalibacterium* can promote the growth of T-regulatory cells or promote the generation of anti-inflammatory cytokines [53]. The gram-positive bacteria called *Romboutsia* appears more prevalent in normal human mucosa and could be connected to host health [54]. Therefore, the members of the genera *Bacteroides*, *Akkermansia, Faecalibacterium* and *Enterococcus* in intestinal are regarded as advantageous microbiota. The high production performance of Taihang chickens was demonstrated by the difference proportion of *Bacteroides*, *Faecalibacterium*, *Enterococcus* and *Romboutsia* in duodenum. As a result, the comparison of the microbiota diversity revealed that several microbes might serve as indicators of excellent production performance.

The LDA score of 4 was utilized as the threshold in the LEfSe analysis to evaluate the differential enrichment of microbial characteristics between H and L groups. This analysis revealed that *Enterococcus* belong to phyla *Firmicutes* was over-presented in H group. The result revealed genera *Enterococcus* as egg production performance biomarker, notably associating Taihang chicken high egg production with the elevated abundance of *Enterococcus*. 

According to microbiota function prediction, the percentage of microbiota functioning in the Metabolism process was the greatest among all differential pathways and was markedly enriched in the H group. Pathways that included Human diseases, environmental information processing, and genetic information processing were more abundant in the L group. Recent reports on the intestinal microbiota of chickens have demonstrated that these microbiotas can produce a variety of enzymes and substrates, which can alter host’s immune system, absorption, and feed efficiency [55,56]. According to metabolism process, the intestinal microbiota may improve feed efficiency, absorption, and immune function in the host, which may be result high performance. On another hand, accept to genetic information, human diseases and environmental information may be the mainly reasons for affecting the performance of Taihang chickens. In conclusion, there were many ways in which the gut microbiota affects the productivity of the host.

## 5. Conclusions

Sequencing of the 16S rRNA amplicon revealed that the intestinal microbiota differed between groups of Taihang chickens with different egg production performance. The composition of duodenal microbes varied between the high and low egg-laying groups. Differences in duodenal microbes and their relationship to metabolism capability have, at least in part, explained their influence on chicken egg-laying performance. These results shed light on how the duodenal microbes influences egg production features and contributions to the establishment of practical strategies for increasing chicken egg production. 

## Figures and Tables

**Table 1:** Composition of the basic diet (dried weight).

Item	Content
Ingredient (%)	
Corn	66.00
Wheat bran	6.80
Soybean meal	21.70
Fish meal	2.0
Dicalcium phosphate	1.20
Limestone	1.30
Premix ^1^	1.00
Total	100
Nutrient composition	
ME (MJ/kg)	11.02
Crude protein (%)	15.76
Calcium (%)	3.32
Available phosphorus (%)	0.32
Total phosphorus (%)	0.57
Lysine (%)	0.72
Methionine (%)	0.34

^1^ Provided per kilogram of diet: Cu (CuSO_4_·H_2_O), 20 mg; Zn (ZnSO_4_·7H_2_O), 70 mg; Fe (FeS-O_4_·H_2_O), 70 mg; Mn (MnSO_4_·5H_2_O), 100 mg; I (KI), 0.4 mg; Se (Na_2_SeO_3_), 0.5 mg; VA, 13,000 IU; VD_3_, 3500 IU; VE, 30 mg; VK_3_, 3 mg; VB_1_, 3 mg; VB_2_, 10 mg; VB_6_, 6 mg; VB_12_, 0.2 mg; pantothenic acid, 10 mg; niacin, 30 mg; folic acid, 0.55 mg; biotin, 0.16; choline chloride, 400 mg.

**Table 2:** Taxonomic assignments of the operational taxonomic units (OTUs).

Taxonomic	Taxonomic Assignment
OTU catalogue	2056
Annotated on database	2055
Annotated Kingdom level	99.95%
Annotated Phylum level	96.50%
Annotated Class level	94.11%
Annotated Order level	88.76%
Annotated Family level	82.98%
Annotated Genus level	57.30%
Annotated Species level	19.16%
Annotated Unclassified:	0.05%

## Data Availability

The 16S rRNA sequencing data can be obtained by contacting the corresponding author.

## Supplementary Materials

The following supporting information can be downloaded at: https://www.mdpi.com/article/10.3390/life12081262/s1, Table S1: Summary statistics of sequences analyzed; Figure S1: The detail sequence reads among samples.
