# Supplementary material for: Microbial Diversity and Community Composition of Duodenum Microbiota of High and Low Egg-Yielding Taihang Chickens Identified Using 16S rRNA Amplicon Sequencing
Source: Life (Basel). 2022 Aug 18;12(8):1262. doi: 10.3390/life12081262 (PMC9409686; doi:10.3390/life12081262)
Supplement: Supplementary file 1 [file life-12-01262-s001.zip › life-1846433-supplementary.pdf]

Table S1. Summary statistics of sequences analyzed.

Sample Name	Raw Reads(#)	Clean Reads(#)	Base(nt)	Avglen(nt)	Q20	GC%	Effective%
H.D.1	60,160	58,086	14,725,532	253	80.75	52.41	96.55
H.D.2	52,765	51,143	12,976,470	253	80.94	52.91	96.93
H.D.3	83,313	80,047	20,311,059	253	88.19	52.39	96.08
H.D.4	53,343	51,668	13,129,322	254	82.31	51.7	96.86
H.D.5	56,944	55,133	14,019,357	254	78.83	52.13	96.82
H.D.6	59,696	57,769	14,704,966	254	79.66	51.77	96.77
H.D.7	76,272	74,361	18,951,190	254	80.28	52.47	97.49
H.D.8	85,163	80,082	20,351,199	254	80.5	52.37	94.03
H.D.9	70,676	67,299	17,064,165	253	83.58	52.54	95.22
H.D.10	87,071	84,185	21,390,156	254	81.98	51.53	96.69
L.D.1	64,293	61,722	15,635,554	253	84.15	51.87	96
L.D.2	77,608	74,353	18,892,663	254	80.91	51.45	95.81
L.D.3	56,145	54,010	13,760,323	254	74.75	52.36	96.2
L.D.4	84,132	80,115	20,421,431	254	81	51.27	95.23
L.D.5	81,747	78,534	19,899,191	253	89.38	51.89	96.07
L.D.6	82,871	80,041	20,376,974	254	82.33	51.93	96.59
L.D.7	82,828	80,074	20,340,505	254	83.94	51.17	96.68
L.D.8	78,228	75,907	19,370,433	255	79.75	51.92	97.03
L.D.9	64,348	62,816	15,924,844	253	85.18	53.48	97.62
L.D.10	56,683	53,996	13,678,740	253	86.26	52.97	95.26
**Average**	**70,714**	**68,067**	**17,296,204**	**253.65**	**82.23**	**52.13**	**96.3**

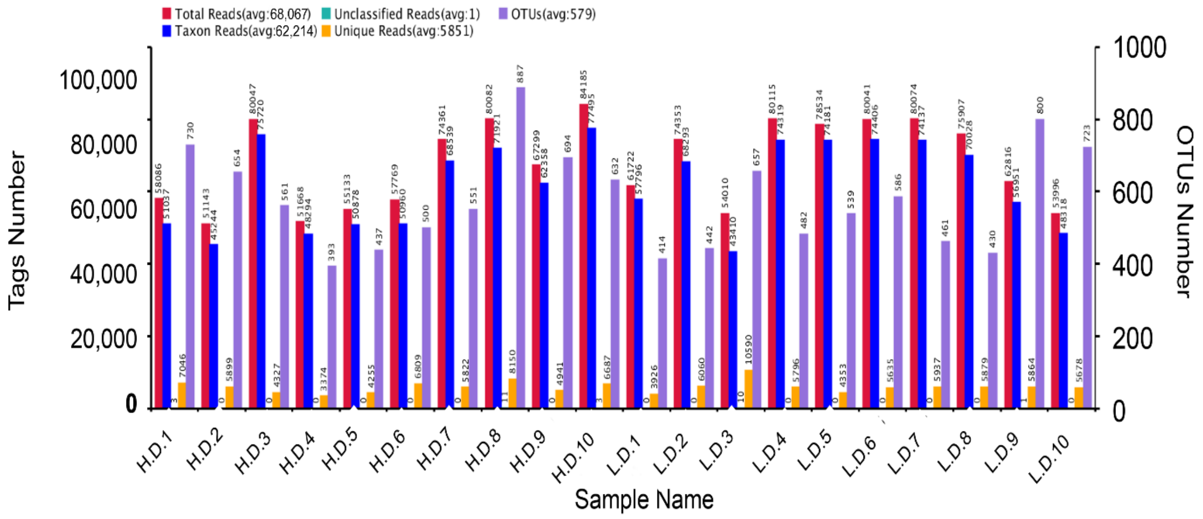

Figure S1. The detail sequence reads among samples.
